# Retinoprotective Effect of 2-Ethyl-3-hydroxy-6-methylpyridine Nicotinate

**DOI:** 10.3390/biology9030045

**Published:** 2020-02-28

**Authors:** Anna Peresypkina, Anton Pazhinsky, Lyudmila Danilenko, Sergey Lugovskoy, Mikhail Pokrovskii, Evgeniya Beskhmelnitsyna, Nikolai Solovev, Anna Pobeda, Mikhail Korokin, Elena Levkova, Victoria Gubareva, Liliya Korokina, Olga Martynova, Vladislav Soldatov, Vladimir Pokrovskii

**Affiliations:** 1 Department of Pharmacology and Clinical Pharmacology, Institute of Medicine, Belgorod State National Research University, Belgorod 308015, Russia; bb_9393@mail.ru (A.P.); danilenko_l@bsu.edu.ru (L.D.); lug90@mail.ru (S.L.); mpokrovsky@yandex.ru (M.P.); evgeny_b89@mail.ru (E.B.); morkovkapro@mail.ru (N.S.); mkorokin@mail.ru (M.K.); last81ya.ru@yandex.ru (E.L.); vikaz@rambler.ru (V.G.); korokina@bsu.edu.ru (L.K.); 2 Research Institute of Pharmacology of Living Systems, Belgorod State National Research University, Belgorod 308015, Russia; martynova_o@bsu.edu.ru (O.M.); zinkfingers@gmail.com (V.S.); 1195606@bsu.edu.ru (V.P.)

**Keywords:** retinal ischemia, electroretinography, laser Doppler flowmetry, ophthalmoscopy, 2-ethyl-3-hydroxy-6-methylpyridine nicotinate

## Abstract

An important task of pharmacology is to find effective agents to improve retinal microcirculation and resistance to ischemia. The purpose of the study is to pharmacologically evaluate the retinoprotective effect of 2-ethyl-3-hydroxy-6-methylpyridine nicotinate in a rat model of retinal ischemia–reperfusion. A retinal ischemia–reperfusion model was used, in which an increase in intraocular pressure (IOP) to 110 mmHg was carried out within 30 min. The retinoprotective effect of 2-ethyl-3-hydroxy-6-methylpyridine nicotinate at a dose of 3.8 mg/kg, in comparison with nicotinic acid at a dose of 2 mg/kg and emoxipine at a dose of 2 mg/kg, was estimated by the changes in the eye fundus during ophthalmoscopy, the retinal microcirculation level with laser Doppler flowmetry (LDF), and electroretinography (ERG) after 72 h of reperfusion. The use of 2-ethyl-3-hydroxy-6-methylpyridine nicotinate prevented the development of ischemic injuries in the fundus and led to an increase in the retinal microcirculation level to 747 (median) (lower and upper quartiles: 693;760) perfusion units (*p* = 0.0002) in comparison with the group that underwent no treatment. In the group with the studied substance, the b-wave amplitude increased significantly (*p* = 0.0022), and the b/a coefficient increased reliably (*p* = 0.0002) in comparison with the group with no treatment. Thus, 2-ethyl-3-hydroxy-6-methylpyridine nicotinate has established itself as a potential retinoprotector.

## 1. Introduction

The retinas have very high oxygen consumption and are sensitive to oxygen deficiency [1,2]. Retinal ischemia is related to the atherosclerosis of retinal vessels and carotid arteries, diabetic retinopathy, glaucoma, and endocrine ophthalmopathy [3,4]. Retinal ischemia leads to an increase in the content of impaired metabolism products which, over time, can lead to atrophy of the optic nerve [5,6]. High intraocular pressure (IOP) cuts the supply of oxygen and glucose, subsequently leading to the induction of apoptosis and retinal cell death [7,8,9,10].

It is necessary to mention the oxidative stress that occurs in the retina during ischemia. Despite a decrease in oxygen concentration as a result of disruption of the electron transport chain of mitochondria, significant amounts of superoxide radicals are released, which are partially reduced to hydrogen peroxide [11]. Then, secondary radicals, such as hydroxyl, are formed, which have powerful destructive potential against cell structures and enzymes [12]. In this regard, the most vulnerable enzymes contain metals with variable valence [13].

As reported by Donato et al., the genes *KLHL7*, *RDH11*, *CERKL*, *AIPL1*, and *USH1G* are proven targets of altered miRNAs: hsa-miR-1307, hsa-miR-3064, hsa-miR-4709, hsa-miR-3615, and hsa-miR-637 respectively. This suggests a connection between oxidative stress and retinitis pigmentosa development. miRNA expression evaluation of oxidative stress-induced retinal pigment epithelium cells has shown a regulative role of miRNA in retinitis pigmentosa that should lead to the discovery of new ways to influence the etiopathogenesis of this disease [14]. Moreover, a decrease in the activity of detected piRNAs could be explained by the inhibition of silencing activity of miRNA regulation of the DNA damage response and transporter activity of retinal pigment epithelium cells in oxidative stress [15].

The search for new biologically active molecules for the prevention and correction of retinal ischemia–reperfusion is one of the tasks of current ocular pharmacology [16,17,18,19].

In this study, the research object was to investigate a new molecule, 2-ethyl-3-hydroxy-6-methylpyridine nicotinate, which includes two pharmacophores: 3-hydroxypyridine and nicotinate. 3-Hydroxypyridine derivatives are used to treat ischemic optic neuropathy. Emoxipine (methylethylpiridinol) is used to treat subconjunctival hemorrhages, intraocular hemorrhages, and angioretinopathy [20]. The presence of 3-hydroxypyridine pharmacophore in a structure provides a complex of antioxidant and membranotropic effects, as well as the reduction of glutamate excitotoxicity [21,22].

Xanthinol nicotinate as a 15% solution for injection is used to treat non-arteritic ischemic optic neuropathy, diabetic angiopathy, and retinopathy [23]. Xanthinol nicotinate dilates peripheral vessels, improves microcirculation in the retinal vessels of the eye, and inhibits platelet aggregation [24,25].

Treatment of ischemic retinal conditions is done using angioprotectors, antioxidants, fibrinolytics, and anticoagulants [21]. Due to the instability effects when carrying out drug therapy, it is necessary to seek out more effective methods to increase resistance to retinal ischemia [26,27,28].

Thus, the relevance of the research is the study of the retinoprotective effects of 2-ethyl-3-hydroxy-6-methylpyridine nicotinate along with the evaluation of the eye fundus state, retinal microcirculation level, and electrophysiological state of the retina, specifically, the b/a coefficient [16,21,29,30] in an experiment when retinal ischemia–reperfusion is corrected.

The purpose of the study is a pharmacological evaluation of the retinoprotective effect of 2-ethyl-3-hydroxy-6-methylpyridine nicotinate in a rat model of retinal ischemia–reperfusion.

## 2. Materials and Methods

### 2.1. Animals

Ethical principles of conducting experiments on laboratory rats were observed in accordance with the European Convention for the Protection of Vertebrate Animals Used for Experimental and Other Scientific Purposes, CETS No. 123. Wistar rats were obtained from the Stolbovaya laboratory animal nursery in the Moscow region, Russia. Experimental studies were conducted on 50 rats (5 groups, 10 animals per group) weighing 225–275 g. For the study, animals with no external signs of disease that had passed the quarantine regime within 14 days were used. All manipulations on rats were performed under general anesthesia with i.p. administration of a solution of chloral hydrate. The experiments were approved by the Local Ethics Committee of Belgorod State National Research University, Belgorod, Russia (Protocol#07/19).

### 2.2. Design of the Experiment

The following groups were included in the experiment: (1) A control group; (2) a group with the retinal ischemia–reperfusion model; (3) a group with correction of retinal ischemia–reperfusion by 2-ethyl-3-hydroxy-6-methylpyridine nicotinate; (4) a group with correction of retinal ischemia–reperfusion by nicotinic acid; and (5) a group with correction of retinal ischemia–reperfusion by Emoxipine.

A retinal ischemia–reperfusion model was used according to the method previously published by us [16,21], in which an increase in IOP to 110 mmHg is carried out within 30 min by applying mechanical pressure to the anterior eye chamber. Retinal ischemia was simulated under general anesthesia (chloral hydrate, 300 mg/kg of rat body mass, i.p.).

**Figure 1:**
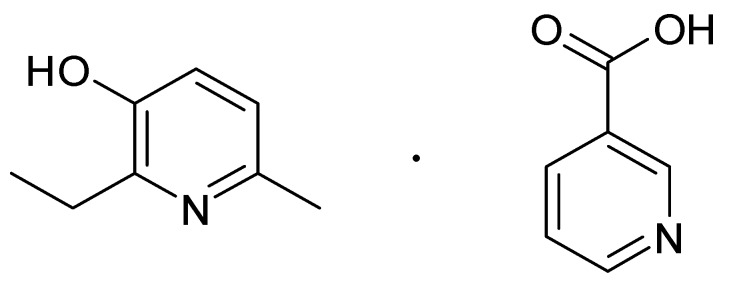
The structure of 2-ethyl-3-hydroxy-6-methylpyridine nicotinate.

The substance 2-ethyl-3-hydroxy-6-methylpyridine nicotinate in the form of a salt was synthesized at the All-Russian Scientific Center for Safety of Biologically Active Substances, Kupavna, Russia. The structure of this substance is shown in Figure 1.

2-Ethyl-3-hydroxy-6-methylpyridine nicotinate was injected parabulbarly as a 1% solution at a dose of 3.8 mg/kg of rat body mass once daily for 4 days, including the first injection 30 min before ischemia–reperfusion modeling. When conducting parabulbar injections, rats were anesthetized (chloral hydrate, 300 mg/kg of rat body mass, i.p.).

Emoxipine (solution for injection, 10 mg/mL, Federal State Unitary Enterprise “Moscow Endocrine Plant”, Moscow, Russia), at a dose of 2 mg/kg [21], was administered in the same manner as 2-ethyl-3-hydroxy-6-methylpyridine nicotinate.

Nicotinic acid (solution for injection, 10 mg/mL, “Novosibkhimfarm” JSC, Novosibirsk, Russia), at a dose of 2 mg/kg, was administered in the same manner as studied substance and Emoxipine. All pharmacological agents were taken for research in equimolar doses (ν = 0.0000146 mol).

In animals of the control group, an equivalent volume of water was injected using the same regime as the studied substance.

The retinoprotective effects of studied pharmacological agents were estimated by changes in the eye fundus during ophthalmoscopy, the retinal microcirculation level with laser Doppler flowmetry (LDF), and electroretinography (ERG) after 72 h of reperfusion [3].

### 2.3. Ophthalmoscopy

Ophthalmoscopy in rats was performed according to the method previously published by us [21,28]. To dilate the pupil, 2.5% Irifrin eye drops (Promed Exports, India) were used. When the eye fundus image was unclear, a proper lens was selected by turning the disk of the ophthalmoscope BXa-13 (Neitz, Japan) to provide a clear image of the eye fundus. An OI-78M lens (Volk Optical Inc, Mentor, OH, USA) was used to magnify and obtain photos of the eye fundus.

### 2.4. Laser Doppler Flowmetry

Seventy-two hours after ischemia simulation, the retinal microcirculation level in rats was measured by LDF according to the previously published method [3]. The online registration was performed using MP150 production Biopac System, Inc. (Goleta, CA, USA), a computer-based data acquisition system with AcqKnowledge 4.2 software, and a TSD-144 needle-type sensor (Biopac System, Inc., Goleta, CA, USA). After the rats were anesthetized (chloral hydrate, 300 mg/kg of rat body mass, i.p.), the microcirculation level was measured at 10 points on the circumference of the eyeball [16].

LDF allows us to evaluate the perfusion parameters of the microcirculation. Probing laser radiation allows one to get a reflected signal from a layer of tissue up to 1 mm thick; the volume of the probed tissue is 1 mm^3^. As the total thickness of the sclera, choroid, and retina in rats is less than 0.5 mm, LDF allows us to assess the level of microcirculation throughout the depth of the probed area.

One perfusion unit is equal to the number of red blood cells passing through 1 mm^3^ of the probed area for 1 s.

### 2.5. Electroretinography

ERG is a tool for fine functional diagnosis, differential diagnosis, and prognosis of retinal and optic nerve diseases and is used to monitor the dynamics of the pathological process and the effectiveness of therapy. ERG was performed in rats according to the method previously published by us [16,21]. The ratio of the amplitudes of the a- and b-waves of ERG, the b/a coefficient, was calculated.

### 2.6. Statistical Analysis

In all cases, the median (Me) and lower (Q_L_) and upper (Q_U_) quartiles were calculated. Between-group differences were analyzed by the Mann–Whitney U test. Statistical analyses were performed using Statistica 10.0 software [31].

## 3. Results

### 3.1. Results of the Eye Fundus State Evaluation

**Figure 2:**
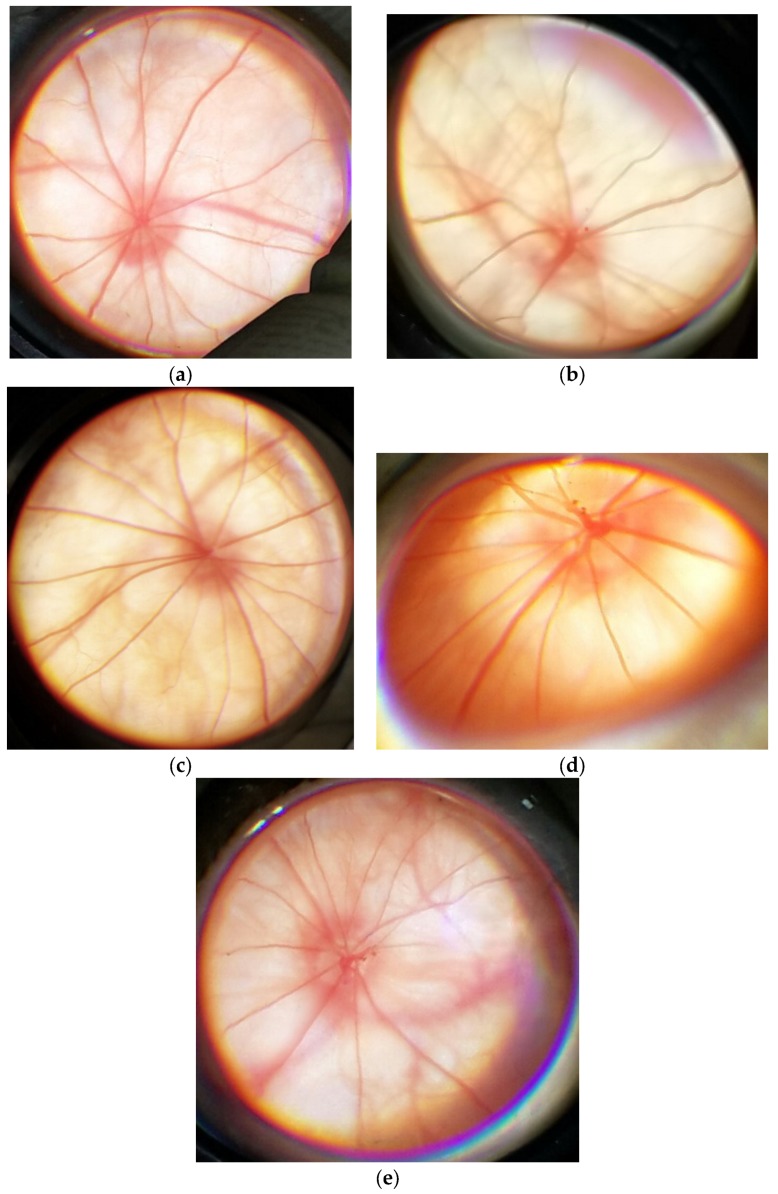
Eye fundus images from rats: (**a**) From the control group; (**b**) with the simulated retinal ischemia–reperfusion; (**c**) with the administration of 2-ethyl-3-hydroxy-6-methylpyridine nicotinate; (**d**) with the administration of nicotinic acid; (**e**) with the administration of Emoxipine.

An example of an eye fundus image from a Wistar rat in the control group is shown in Figure 2a and can be considered the norm.

An example of the eye fundus image of a rat with the simulated retinal pathology is shown in Figure 2b and can be characterized in this way: The optic disc (OD) is partially decolorated and edematous. The boundaries of the OD are not well-defined. The arteries are spasmed; the veins are dilated. There are spot hemorrhages above the surface of the OD. The eye fundus background is pallid.

When 2-ethyl-3-hydroxy-6-methylpyridine nicotinate was injected at a dose of 3.8 mg/kg, the correction of the retinal vessels’ caliber was observed, but the veins were dilated. No hemorrhages were observed. The OD was pink. Its boundaries were well-defined (Figure 2c).

When nicotinic acid was injected at a dose of 2 mg/kg, the eye fundus image was as follows: The OD is partially decolorated. There are spot hemorrhages over the OD. There are no spasms of the arteries, but the veins are full-blooded (Figure 2d).

When Emoxipine was injected at a dose of 2 mg/kg to correct the simulated pathology, the fundus image was as follows: This OD is partially decolorated. There are spot hemorrhages over the OD. The retinal arteries are spasmed (Figure 2e).

In monotherapy with Emoxipine or nicotinic acid, there were no improvements in the state of the OD, and no resorption of hemorrhages over the OD was observed. Administration of nicotinic acid led to the correction of the caliber of retinal arteries. When 2-ethyl-3-hydroxy-6-methylpyridine nicotinate was administered, the correction of both pathological vascular changes and the state of the OD were observed.

### 3.2. Results of the LDF

**Figure 3:**
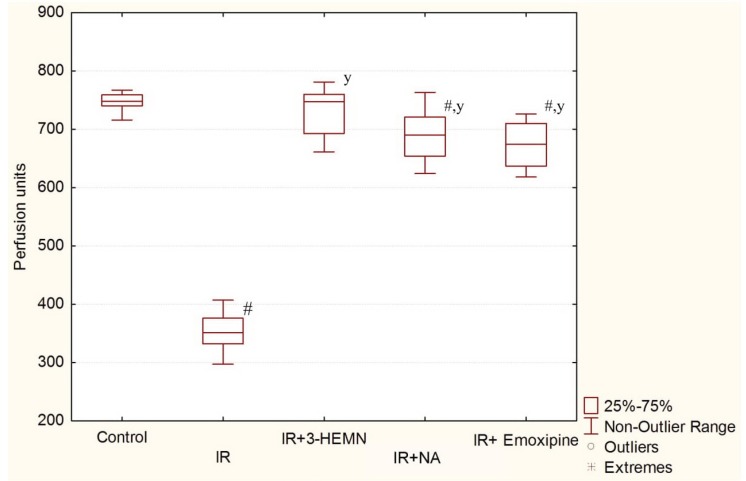
Retinal microcirculation level in experimental groups (*n* = 10). IR: group with retinal ischemia–reperfusion; IR+3-HEMN: group with retinal ischemia–reperfusion treated with 2-ethyl-3-hydroxy-6-methylpyridine nicotinate; IR+NA: group with retinal ischemia–reperfusion treated with nicotinic acid; # *p* = 0.0002 compared to the control (for IR group); ^y^ *p* = 0.0002 compared to the ischemia–reperfusion model (for the IR+3HEMN group); # *p* = 0.0065 compared to the control (for the IR+NA group); ^y^ *p* = 0.0002 compared to the ischemia–reperfusion model (for the IR+NA group); # *p* = 0.0002 compared to the control (for the IR+Emoxipine group); ^y^ *p* = 0.0002 compared to the ischemia–reperfusion model (for the IR+Emoxipine group).

The results of the evaluation of the retinal microcirculation using LDF are presented in Figure 3.

In the group with simulated pathology, the microcirculation level was 352 (median; Q_L_ = 332; Q_U_ = 376) perfusion units, which was significantly different (*p* = 0.0002) from the control group. The use of 2-ethyl-3-hydroxy-6-methylpyridine nicotinate led to an increase in the microcirculation level to 747 (median; Q_L_ = 693; Q_U_ = 760) perfusion units (*p* = 0.0002), in comparison with the group with retinal ischemia–reperfusion, and the values of the control group were reached. In the group with nicotinic acid, the microcirculation level was 690 (median; Q_L_ = 654; Q_U_ = 721) perfusion units, which was significantly different (*p* = 0.0065) from the control. In the group with Emoxipine, the retinal microcirculation level was 674 (median; Q_L_ = 637; Q_U_ = 710) perfusion units, which was also significantly different (*p* = 0.0002) from the control. Thus, correction of the microcirculation level in the retina by 2-ethyl-3-hydroxy-6-methylpyridine nicotinate was more pronounced than when Emoxipine and nicotinic acid were used as monotherapies in the retinal ischemia–reperfusion model.

### 3.3. Results of the ERG

**Figure 4:**
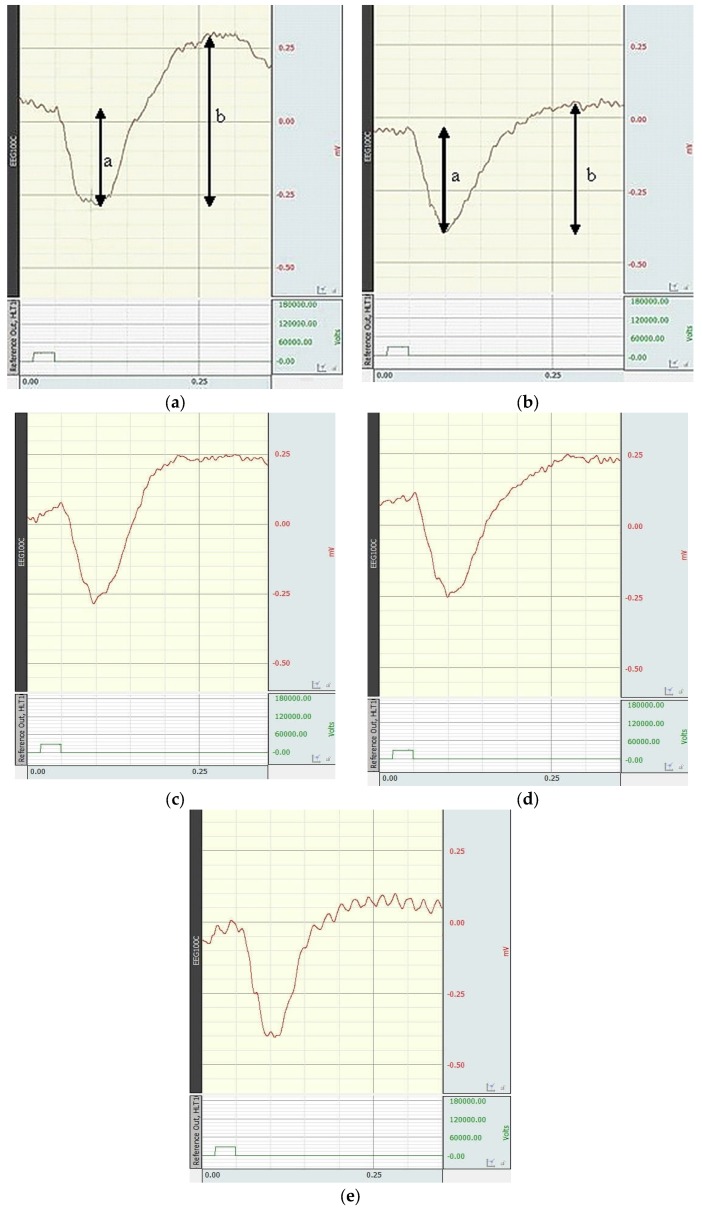
Electroretinograms of Wistar rats: (**a**) Control group; (**b**) with the simulated retinal ischemia–reperfusion; (**c**) with the administration of 2-ethyl-3-hydroxy-6-methylpyridine nicotinate; (**d**) with the administration of nicotinic acid; (**e**) with the administration of Emoxipine. Arrows show the amplitudes of the a-wave and b-wave.

Examples of electroretinograms of Wistar rats in experimental groups are presented in Figure 4. When simulating retinal ischemia–reperfusion, the amplitude of the b-wave changed significantly, the suppression of which is shown in Figure 4b. When correcting the simulated pathology by the studied substance, nicotinic acid, and Emoxipine, an increase in the b-wave amplitude to varying degrees was observed (Figure 4c–e).

The influences of the studied substance, nicotinic acid, and Emoxipine on the a- and b-wave amplitudes and the b/a coefficient when correcting retinal ischemia–reperfusion are presented in Table 1.

Significant changes in the a-wave amplitude were not observed in animals with retinal ischemia–reperfusion and animals treated with pharmacological agents. When simulating pathology, the amplitude of the b-wave reduced by more than 2 times (*p* = 0.0009) in comparison with the control. In the group treated with the studied substance, the b-wave amplitude increased significantly (*p* = 0.0022) in comparison with the group with no treatment; the b/a coefficient increased reliably (*p* = 0.0002) in comparison with the group with no treatment. As can be seen from the table data from the ERG study, the best correction of ischemic retinal damage was found in 2-ethyl-3-hydroxy-6-methylpyridine nicotinate.

Thus, the results of ophthalmoscopy, LDF, and ERG after 72 h of reperfusion in the retinal ischemia–reperfusion model showed the most pronounced retinoprotective effect of 2-ethyl-3-hydroxy-6-methylpyridine nicotinate at a dose of 3.8 mg/kg of rat mass.

## 4. Discussion

The detected retinoprotective action of 2-ethyl-3-hydroxy-6-methylpyridine nicotinate at a dose of 3.8 mg/kg in rats to correct retinal ischemia–reperfusion may be associated with following: (a) nicotinate is a “scavenger” of the superoxide radical from which other radicals, such as hydroxyl, which have a powerful destructive potential against cell structures and enzymes, are subsequently formed [32]; (b) nicotinate dilates peripheral vessels, improving microcirculation in the retinal vessels [24]; and (c) 3-hydroxypyridine is a “scavenger” of radical oxidation products, such as peroxides and superoxides of fatty acids [32].

“Vitamin B3”, or “vitamin PP”, includes two vitamers (nicotinic acid and nicotinamide), giving rise to the coenzymatic forms nicotinamide adenine dinucleotide (NAD) and nicotinamide adenine dinucleotide phosphate (NADP). A growing body of evidence highlights the key role of vitamin B3 in neuronal health. What is emerging is that niacin bioavailability is crucial for neuron survival and functions. Indeed, vitamin deficiency has been recognized as a pathogenic factor for neurological deficits and dementia, as well as for neuronal injury and psychiatric disorders [33].

CNS vascular integrity is connected with the NAD content in the brain [34]. Zhang et al. noted that heterozygous deletion of nicotinamide phosphoribosyltransferase (NAMPT) in the brain exacerbates focal ischemic stroke-induced neuronal death and brain damage [35], while its selective knockdown in projection neurons of adult mice leads to motor dysfunction, neurodegeneration, and death [36].

Fundamental discoveries accentuate a therapeutic potential for targeting NAD+ biosynthetic pathways in the treatment of conditions such as ischemia and hypoxia [37]. The research of NAD+ has been reactivated by new knowledge about the dynamics within NAD+ metabolism that trigger major signaling processes coupled to sirtuins, PARPs, and CD38 and redirect the vector of cell metabolism [38]. Cellular regulation includes the activation of mitochondrial biogenesis, a process fundamental to physiological adjustment to ischemia. Several metabolic pathways converge to NAD+, including tryptophan-derived de novo pathways, nicotinamide salvage pathways, and nicotinic acid salvage and nucleoside salvage pathways, incorporating nicotinamide riboside and nicotinic acid riboside [39]. NAD is a substrate of ADP-ribosyltransferases that catalyze ADP-ribose transfer reactions, thus breaking down NAD to nicotinamide and ADP-ribosyl products, which play key roles in cellular signaling cascades by regulating gene expression, cell cycle progression, insulin secretion, DNA repair, apoptosis, and aging [40].

Possibly, the pronounced retinoprotective effect of the substance studied in the experiment is associated with an increase in the antioxidant and neuroprotective properties of two pharmacophores and an improvement in the microcirculation in the retina.

Currently, a large amount of research has been devoted to the search for, and study of, pharmacological agents with antioxidant effects for the correction of retinal ischemic injury [41,42,43]. According to some authors, during the early phase of an ischemic attack, an increase in extracellular glutamate activates N-methyl-D-aspartate (NMDA) receptors, causing a Ca^2+^ influx due to activated NMDA receptors. In addition, nitric oxide (NO) is produced under the action of nitric oxide synthase in the mitochondria. NO and O^2−^ combine to produce the highly reactive species peroxynitrite (ONOO^−^) [44,45].

Based on the literature data, 3-hydroxypyridine provides a reduction in glutamate excitotoxicity [22]. Therefore, a study of the effect of 2-ethyl-3-hydroxy-6-methylpyridine nicotinate on the model of NMDA-induced excitotoxicity in the retina is planned to confirm the intended biological target.

Howell et al. reported that inflammation is an important phenomenon in ischemia-induced cellular apoptosis, and there is increasing evidence to show that neuroinflammation plays an important role in the glaucomatous loss of retinal ganglion cells [46]. Yoneda et al. reported ischemia-induced increased Interleukin (IL)-1β mRNA expression in the retina from 3 to 12 h after reperfusion [47]. Interleukin (IL)-1β and tumor necrosis factor (TNF)-α have been shown to cause the induction of iNOS, leading to increased nitrosative and oxidative stress [48].

Retinal ischemia leads to a depletion of ATP and subsequent loss of ionic homeostasis and depolarization. Uncontrolled depolarization activates the self-reinforcing excitoxicity cascade [49].

In view of the above, the study of possible ways to correct retinal ischemia, which accompanies a number of eye pathologies, is an important task for ocular pharmacology.

## 5. Limitations

Ischemia leads to the inhibition of metabolic processes in the retina with cellular apoptosis [9]. Therefore, we plan to investigate possible antiapoptotic mechanism of 2-ethyl-3-hydroxy-6-methylpyridine nicotinate, specifically, p53 activity, Bcl-2, caspase-3, and Nf-kB activity in the correction of retinal ischemia–reperfusion and NMDA-induced excitotoxicity in the retina.

## 6. Conclusions

2-Ethyl-3-hydroxy-6-methylpyridine nicotinate, at a dose of 3.8 mg/kg of rat mass, has a pronounced retinoprotective effect, which is superior to the effects of nicotinic acid and Emoxipine monotherapies in equimolar doses in a model of retinal ischemia–reperfusion. This was expressed as improvement in eye fundus images (as the alignment of the retinal vessel caliber and the prevention of hemorrhages), the attainment of target values of the microcirculation level in the retina, and improvement in the electrophysiological state of the retina.

## Figures and Tables

**Table 1:** Influences of 2-ethyl-3-hydroxy-6-methylpyridine nicotinate, nicotinic acid, and Emoxipine on the a- and b- wave amplitudes (mV) and the b/a coefficient when correcting retinal ischemia–reperfusion (Me (Q_L_;Qu)).

Groups of Animals	The a-wave Amplitudes (*n* = 10)	The b-wave Amplitudes (*n* = 10)	The b/a Coefficient (*n* = 10)
Control	0.36 (0.29;0.42)	0.98 (0.67;1.07)	2.60 (2.28;2.77)
Retinal ischemia–reperfusion	0.36 (0.29;0.47)	0.42 (0.33;0.52) #	1.19 (1.10;1.32) #
Retinal ischemia–reperfusion + 2-ethyl-3-hydroxy-6-methylpyridine nicotinate 3.8 mg/kg	0.33 (0.27;0.47)	0.83 (0.76;1.01) ^y^	2.25 (2.09;2.69) ^y^
Retinal ischemia–reperfusion + Nicotinic acid 2 mg/kg	0.37 (0.28;0.48)	0.77 (0.57;0.95) ^y^	2.06 (1.93;2.16) #^y^
Retinal ischemia–reperfusion + Emoxipine 2 mg/kg	0.32 (0.27;0.50)	0.77 (0.57;1.01) ^y^	2.18 (2.00;2.33) #^y^

# compared to the control; ^y^ compared to the ischemia–reperfusion model.
